# Associations of Gaze and Facial Behaviors with Subjective Perceptions of Facial Expressions in a Human-like Android Robot

**DOI:** 10.3390/biomimetics11100702

**Published:** 2026-10-02

**Authors:** Ayaka Fujii, Bowen Wu, Takashi Minato

**Affiliations:** Guardian Robot Project, RIKEN, Kyoto 619-0288, Japan; bowen.wu@riken.jp (B.W.); takashi.minato@riken.jp (T.M.)

**Keywords:** human–robot interaction, facial expression, gaze, android robot

## Abstract

Android robots with human-like faces have been developed based on biomimetic principles to reproduce human facial characteristics and expressive behavior. However, the same facial expression may be perceived differently depending on individual perceptual tendencies and conversational context. This study examined whether such subjective evaluations of android facial expressions are reflected in observers’ gaze and facial responses during face-to-face dialogue. Fourteen adults conversed with an android robot and evaluated each facial expression in terms of contextual strangeness and familiarity. Wearable eye tracking was used to measure gaze allocation across facial regions, while participants’ facial movements were extracted from video. Behavioral responses during the 2.5 s following each of the android’s facial-expression events were analyzed. Expressions perceived as strange in the conversational context were associated with reduced mouth-directed gaze and greater activation of several mouth-related blendshapes compared with those perceived as not strange. For unfamiliar expressions, gaze shifted away from the eyes and toward areas outside the face around 1 s after the expression command, whereas familiar expressions showed the opposite pattern. These exploratory findings suggest that subjective perceptions of android facial expressions are reflected in both gaze allocation and facial behavior during interaction.

## 1. Introduction

In face-to-face human communication, the face serves as an important nonverbal channel through which people convey cues relevant to affective states, communicative intentions, mutual understanding, and social interaction [1,2,3]. Facial expressions also play an important role in social robots that work in applications such as daily-life assistance and customer service, where they continuously interact with people. Such robots are expected to behave appropriately and convey their affective states through nonverbal cues [4,5,6].

Among such robots, androids with human-like faces have been developed based on biomimetic approaches that reproduce human facial structure and movement to realize human-like facial expressions [7,8,9,10,11,12,13,14,15]. Research on android facial expressions has broadly taken two approaches. The first approach concerns morphological and kinematic validity, which refers to whether an android can mechanically reproduce human facial movements, such as those represented using Action Units (AUs) in the Facial Action Coding System (FACS) [16,17] or facial blendshapes [18]. The second approach concerns perceptual validity, which refers to whether the generated expressions are recognized as conveying the intended emotions and are perceived as natural and appropriate.

However, although facial expressions show some common perceptual patterns across individuals, individual differences in their recognition and interpretation have been reported [19,20]. Consequently, the same facial expression may be judged differently by different people. Such variability has been observed in evaluations of both human and android facial expressions, where recognition and interpretation show considerable but incomplete agreement across all observers [13,21].

Rather than treating such variability as mere deviation, it may provide useful information for adapting robot behavior during interaction. If individual differences in facial expression perception are reflected in users’ nonverbal behaviors, robots could use these behavioral cues to adapt their facial expressions, potentially contributing to more effective human–robot interaction (HRI). To realize such adaptation, it is first necessary to understand whether and how differences in facial-expression perception are reflected in users’ nonverbal responses.

Previous studies have investigated human responses to android facial expressions and reported facial muscle activity and observable facial movements associated with the emotional categories of the displayed expressions [22,23]. However, many of these studies have relied on a small, predefined set of prototypical emotional expressions presented under controlled conditions. Although such designs are useful for isolating specific aspects of human responses, they do not capture the broader, context-dependent variety of facial expressions that emerges during ongoing conversation. It therefore remains unclear what facial responses humans exhibit to dynamically generated robot expressions during real-time face-to-face interaction.

In addition to facial responses, gaze behavior has been widely examined as an indicator of human responses during HRI. Human gaze behavior is influenced by the robot’s gaze [24], and gaze toward the robot has been associated with subjective impressions of the robot [25]. Together, these findings suggest that both facial and gaze behaviors can provide information about how humans respond to robots. However, little is known about how these behaviors relate to subjective evaluations of individual facial expressions during ongoing interaction.

Accordingly, this study aims to clarify how subjective perceptions of facial expressions performed by an android during face-to-face interaction are reflected in the human’s gaze and facial behaviors. In particular, we focus on whether perceived strangeness and familiarity of facial expressions are reflected in nonverbal behavior, and address the following research questions.

**RQ1.**  *How are subjective evaluations of an android’s facial expressions reflected in humans’ gaze allocation across facial regions?*

**RQ2.**  *How are subjective evaluations of an android’s facial expressions reflected in humans’ own facial behaviors?*

The main contributions of this study are twofold.

1. Rather than using a small predefined set of facial expressions as stimuli, we investigate human responses to facial expressions performed in real time by an android robot during face-to-face conversation.
2. By relating subjective evaluations of the robot’s facial expressions to human gaze and facial responses, we examine how perceptual differences are reflected in human behavior.

The findings of this study are expected to provide a foundation for future adaptive HRI systems in which a robot adjusts its facial expressions based on the responses of individual interaction partners.

The remainder of this paper is organized as follows. Section 2 reviews related work on human-like facial expressions in robots and human nonverbal behavior, and positions this study within this research context. Section 3 describes the experimental system, procedure, and data analysis methods. Section 4 presents the results of the gaze and facial behavior analyses, and Section 5 discusses the findings and limitations of the study. Finally, Section 6 concludes the paper.

## 2. Related Work

### 2.1. Human-like Facial Expression Generation and Evaluation in Robots

#### 2.1.1. Facial Expression Implementation Based on Human Facial Principles

Reproducing human-like facial expressions on robots requires a systematic representation of human facial movements and their mechanical implementation. The FACS describes observable facial movements in terms of anatomically based AUs [16,17], providing a common framework for describing and comparing facial expressions in humans and android robots. Related representations such as facial blendshapes have also been used to describe facial deformations and map human facial movements onto robotic faces [26].

These representations have been incorporated into the design and control of android facial mechanisms. For example, the facial actuation points of the android robot Yui were designed based on AUs, with facial movements mapped to corresponding actuators [15]. Other studies have used blendshape representations to retarget human facial expressions to robotic faces by modeling the mapping between human facial movements and robot actuation [27]. Such approaches provide a systematic link between human and android facial behavior.

#### 2.1.2. Perceptual Evaluation of Facial Expressions

Robot facial expressions implemented based on human facial principles have been evaluated in terms of whether they can convey intended emotions and whether the expressions are perceived as natural and human-like. Many human-like robots have been shown to produce distinct facial expressions for multiple basic emotions, including anger and sadness [8,13,28,29,30].

The perception of a robot’s facial expression also depends on the conversational context in which it is performed. Bennett et al. showed that robotic facial expressions were recognized more accurately when they were congruent with the emotional context [31]. In a dialogue study with Furhat, context-congruent emotional expressions were perceived as more emotionally appropriate and more human-like, and elicited a more positive impression than incongruent or neutral expressions [32]. These studies suggest that impressions of robot facial expressions depend not only on their facial configuration and recognizability, but also on the context in which they are expressed.

### 2.2. Analyses of Human Nonverbal Behaviors

#### 2.2.1. Gaze Behavior

Gaze in interaction is not merely an indicator of where a human directs attention. Perugia et al. found that gaze toward the robot during social conversation was related to subjective evaluations such as uncanniness, likability, and warmth [25]. Thepsoonthorn et al. found that participants’ fixation duration toward a robot varied with perceived human-likeness and affinity [33].

Human gaze behavior can also be influenced by the robot’s behavior. Xu et al. found that when the robot looked at the participant’s face more frequently, participants looked back at the robot more often, consequently increasing mutual gaze [24]. Song et al. found that human visual attention varied with the emotional expressions performed by a robot, with positive facial expressions attracting more attention than negative expressions [34].

These findings suggest that human gaze behavior during interaction reflects both how the robot is perceived and how humans respond to the robot’s behavior.

#### 2.2.2. Facial Behavior During Observation of Facial Expressions

Humans are known to exhibit rapid facial responses when observing others’ facial expressions. When participants viewed dynamic expressions of happiness and anger, corresponding facial actions, such as lip-corner pulling and brow lowering, were observed shortly after the facial change [35]. Such responses are also influenced by how facial expressions are presented. Dynamic expressions have been shown to elicit clearer facial mimicry than static expressions, while live expressions can evoke stronger facial responses than prerecorded expressions [35,36].

Similar facial behaviors have also been observed when humans view facial expressions performed by robots. Studies with android robots have shown that robot facial expressions can elicit corresponding facial responses in human observers, as measured by electromyography and video-based facial analysis [22,23]. These findings suggest that human-like facial expressions produced by robots can elicit spontaneous facial behavior in humans.

### 2.3. Positioning of This Study

Previous studies have established that gaze and facial behaviors can provide observable indicators of human responses to robots. However, these studies have mainly examined responses to predefined robot behaviors or broad subjective impressions of the robot, rather than how people respond to individual facial expressions during ongoing interaction. In particular, it remains unclear whether differences in the subjective perception of a facial expression are reflected in the gaze and facial behaviors that immediately follow that expression.

This study addresses this gap by relating subjective evaluations of individual facial-expression events to the subsequent gaze and facial behaviors of participants. The aim of this study is not to test whether specific facial expressions causally elicited particular responses in participants. Rather, we examine whether perceived strangeness and familiarity of android facial expressions are associated with differences in gaze allocation across robot facial regions and in participants’ facial movements.

## 3. Materials and Methods

### 3.1. Overview of the Methods

#### 3.1.1. Study Design

In this study, we conducted a face-to-face interaction experiment in which participants freely conversed with an android robot capable of performing human-like facial expressions while participants’ gaze and facial behavior were recorded. During the interaction, the android robot displayed facial expressions selected in real time by the system according to the conversational context.

The basic unit of analysis was a “facial-expression event,” defined by the time at which a facial-actuator command was sent to the android. For each facial-expression event, we linked (1) the participant’s retrospective subjective evaluation of the corresponding expression, (2) gaze position obtained from the wearable eye tracker worn by the participant, and (3) facial blendshape values (ranging from 0 to 1, with higher values indicating a greater degree of the corresponding facial movement) extracted from video recordings of the participant. This design allowed us to examine within-participant differences in the evaluation of individual facial-expression events and their relationships with subsequent behavioral responses, rather than relying only on post-interaction impressions of an entire dialogue session. An overview of the study design is shown in Figure 1.

#### 3.1.2. Data Collection and Analysis Flow

Participants engaged in free conversation with the android robot for approximately 5 min per session. During each interaction, the participant’s gaze, facial video, dialogue audio, and facial-expression commands sent to the android were synchronously recorded. Immediately after each dialogue session, participants reviewed the recorded interaction and evaluated, in chronological order, each facial expression displayed by the android during the conversation. Two dialogue-and-evaluation sessions were planned for each participant. However, because of system problems and experimental time constraints, a total of 24 dialogue sessions were ultimately collected from 14 participants.

For the behavioral analyses, the time at which the facial-expression command was sent was defined as time 0, and the following 2.5 s were analyzed. This interval was divided into five 0.5-s time windows: 0–0.5 s, 0.5–1.0 s, 1.0–1.5 s, 1.5–2.0 s, and 2.0–2.5 s. For each time window, we calculated the proportion of gaze duration directed toward the robot’s facial regions, particularly the eyes and mouth, and the mean facial blendshape values of the participant. The gaze analysis included facial-expression events that occurred while the robot was either speaking or listening. The facial analysis was restricted to events that occurred while the robot was speaking in order to reduce the influence of the participant’s own speech-related mouth movements.

ChatGPT (GPT-5.6 Sol, OpenAI, San Francisco, CA, USA) was used as an assistive tool for the development and refinement of software and data-analysis scripts, as well as for drafting portions of the manuscript and translating text into English. All AI-assisted code and text were reviewed and edited by the authors.

### 3.2. Android Robot Nikola

The android Nikola (RIKEN, Kyoto, Japan) served as the conversational partner. Nikola has a human-like facial morphology and is covered with soft silicone skin over the frontal face and neck [13]. It is equipped with 35 pneumatic actuators: 29 for facial movements, three for eye movements, and three for head/neck movements. The facial actuation mechanism was designed to enable movements of the eyebrows, eyelids, eyeballs, cheeks, mouth, and neck. In addition to basic emotional expressions, Nikola can generate a broad range of facial expressions representing complex emotions and physical states that may occur during conversation [37,38,39]. Examples of Nikola’s various facial expressions are shown in Figure 2.

### 3.3. Dialogue System

#### 3.3.1. Overview of the Dialogue System

The Realtime API (OpenAI) was used as the basis of the dialogue system [40]. The same audio stream of the participant’s speech was sent in parallel to two independent Realtime API modules. The first was the main dialogue module (gpt-realtime-2), which generated the robot’s verbal response, selected the facial-expression category used while the robot was speaking, and determined emotional parameters for speech synthesis. The second was an emotion-estimation module (gpt-realtime-mini), which estimated affective states in valence–arousal (VA) space and determined the facial-expression category used while the robot was listening.

Server-side voice activity detection (server VAD) in the Realtime API was used to detect participant speech segments. The VAD parameters were empirically adjusted to support natural turn-taking. The silence duration used for end-of-speech detection was set to 300 ms for the main dialogue module and 100 ms for the emotion-estimation module. The longer threshold in the main dialogue module reduced the likelihood that short within-utterance pauses would be incorrectly treated as the end of a turn, whereas the shorter threshold in the emotion-estimation module enabled changes in speech content to be processed with lower latency and at a finer temporal granularity. In addition, when new participant speech was detected while the main dialogue module was generating a response, the ongoing response generation was interrupted and a new response was generated using the latest participant utterance. These mechanisms were intended to preserve response immediacy while allowing the system to incorporate the participant’s most recent speech.

In addition to the participant’s streaming audio, both modules received the dialogue history and state information indicating whether the robot was speaking or listening. The main dialogue module also continuously received the most recent valence and arousal values output by the emotion-estimation module.

#### 3.3.2. Response Generation Using Generative AI

The outputs of both modules were structured as JSON objects conforming to predefined function-call schemas. This allowed downstream processes to access model outputs in a consistent format. Although the Realtime API supports audio response generation, only text output was used in this study because the subsequent robot-control processes required structured information about the robot’s verbal and nonverbal responses.

The structured output of the main dialogue module included the robot’s response text, candidate facial-expression categories, gaze direction, and speech-expression parameters. For facial-expression categories, the module output one to five candidates selected from the 75 expression categories defined in our previous facial-expression database [39], together with a neutral category. Gaze direction was selected from four options: looking straight ahead, looking laterally or diagonally away, looking downward, and looking upward. Speech was synthesized using AITalk (AI, Inc., Tokyo, Japan), and the main dialogue module output the speech-expression parameters corresponding to joy, anger, and sadness.

The response text was generally limited to one or two sentences. For two-sentence responses, separate facial-expression and speech-expression parameters were output for each sentence when a change between the sentences was required. Otherwise, a single set of parameters was used for the entire response. Each resulting text–parameter unit was treated as one utterance and added sequentially to the robot’s speech queue.

In the emotion-estimation module, affective states were represented in valence–arousal space, with both dimensions ranging from −1 to 1. Valence represented affect from unpleasant (−1) to pleasant (1), whereas arousal represented activation from low arousal (−1) to high arousal (1). The structured output included estimated valence and arousal values for the robot’s internal affective state and target valence and arousal values for the affect that the robot should externally express (target expression VA). The output also included candidate facial-expression categories and gaze direction, using the same formats as those of the main dialogue module.

For facial-expression category and gaze-direction control, the output of the main dialogue module was used while the robot was speaking, whereas the output of the emotion-estimation module was used while the robot was listening. Therefore, expressions during robot speech were selected to correspond to the generated verbal response, while expressions during listening could be updated according to continuously estimated affective states and conversational content.

#### 3.3.3. Selection and Presentation of Facial Expressions Using the Expression Database

The facial expression performed by the robot was determined by matching the candidate facial-expression categories, gaze direction, and target expression VA against a facial-expression database for Nikola. The database used in the interaction system was constructed based on our previous work [39] and comprised 676 Nikola expressions across 76 categories, including a neutral category. Each category contained multiple facial expressions with variations in facial configuration. Each expression was associated with one of four gaze directions: straight ahead, lateral/diagonal, upward, or downward. The neutral category contained four expressions, one for each gaze direction, whereas each of the other 75 categories contained five to ten expressions. The available gaze directions varied across categories, and not all four directions were represented in every category.

Within the same category, differences in facial configuration could lead to variation in valence and arousal values across expressions. For the neutral expressions, both valence and arousal were set to 0. For expressions in the other 75 categories, valence and arousal values were estimated individually from the corresponding facial images using GPT-5.2 (OpenAI).

For facial-expression selection, the system first retrieved expressions from the database that belonged to the expression-category candidates and matched the specified gaze direction. Next, the squared Euclidean distance between the VA values assigned to each candidate expression and the target expression VA was calculated. The expression with the minimum distance was selected as the final expression. If no expression with the specified gaze direction was available for a candidate category, an expression with a straight-ahead gaze from the same category was used instead.

During interaction, gaze aversion was also used as a turn-taking cue. Previous work has shown that gaze direction helps coordinate turn transitions and that gaze aversion during pauses can signal continued turn holding [41,42]. Accordingly, when unpresented utterances remained in the robot’s speech queue, the gaze direction was set to lateral or diagonal to indicate that the robot was still holding the conversational turn during temporary pauses.

While the robot was speaking, its facial expression was updated at the onset of each utterance. Lip synchronization was generated using in-house software. Consequently, although the selected expression was presented at utterance onset, the shape of the mouth changed during the utterance in synchrony with the synthesized speech.

While the robot was listening, the facial expression was updated when a new output from the emotion-estimation module became available. To avoid unnaturally frequent expression changes, a minimum interval of 2 s was imposed between expression updates during listening.

#### 3.3.4. Modules for Natural Face-to-Face Interaction

To support natural face-to-face interaction, several modules for generating listener behaviors were incorporated in parallel with facial expression generation.

MaAI, a real-time behavior-prediction module based on speech information, was used to generate listener behaviors such as nodding and backchannels [43,44,45]. It received two audio channels, one from the participant and one from the robot, and continuously predicted the types and timing of backchannels and nods, which were then reflected in the robot’s behavior.

For verbal backchannels, multiple utterances covering a range of valence and arousal levels were recorded in advance. The presented backchannel was selected according to both the backchannel type predicted by MaAI and the current valence and arousal state. Nods were generated according to MaAI predictions and were additionally triggered when the main Realtime API dialogue module detected the end of a participant utterance. To avoid repeated responses within short periods, minimum intervals of 2 s for nods and 3 s for verbal backchannels were imposed.

In addition, we generated continuous neck movement for the robot during its listening phase by using DualTalk [46], which is a deep learning-based model trained on human dyadic conversation to generate neck motions and facial expressions for either speaker, conditioned on the speaker’s own speech, along with the interlocutor’s speech, neck motions, and facial expressions. In our system, the same model used the robot’s own speech along with the participant’s multimodal inputs to generate neck motions and facial expressions. We only used the head motion in the output to control the robot’s neck, by transforming and constraining the output values to match the robot configuration.

### 3.4. Measurement Devices

Participants’ gaze behavior was measured using a Tobii Pro Glasses 2 eye tracker (Tobii AB, Stockholm, Sweden). The recorded data included a scene video from the participant’s first-person viewpoint, two-dimensional gaze coordinates, and timestamps corresponding to each gaze sample. Gaze data were recorded at 100 Hz, while the scene video was recorded at 25 fps. Participants’ facial movements were captured in RGB video at 30 fps using an Azure Kinect DK (Microsoft Corporation, Redmond, WA, USA) placed next to the robot.

### 3.5. Experimental Procedure

Before the experiment, participants received an explanation of the study, and informed consent was obtained from all participants. After providing consent, participants completed a pre-experiment questionnaire concerning basic demographic information and related background information. Participants then wore the eye tracker, and calibration was performed. After preparation was complete, the interaction with Nikola began.

Participants engaged in approximately 5 min of free conversation with Nikola in each session. In the first session, Nikola gave a self-introduction and asked the participant what they had eaten for dinner the previous day. The conversation then proceeded according to the participant’s responses. In the second session, Nikola asked the participant what topic they would like to talk about, and the subsequent conversation proceeded freely from that response. Neither the dialogue content nor the facial expressions to be presented were predetermined, and they were generated in real time according to the ongoing interaction. Participants were not instructed to pay particular attention to specific facial expressions or facial regions.

Immediately after each dialogue session, participants retrospectively evaluated each facial expression displayed by Nikola while reviewing the recorded interaction using an in-house evaluation interface. The interface presented video and audio recorded by a camera positioned above and behind the participant, the first-person scene video recorded by the eye tracker, and the image from the facial-expression database corresponding to the expression that had been presented. Given the communication latency with the robot and the time required for the pneumatic actuators to reach the commanded state and complete the facial expression, the evaluation time point for each expression event was set to 0.5 s after transmission of the facial-expression command.

For each expression event, participants answered the following questions:

**Q1.**  *“Do you think this facial expression is strange in this conversational context?”*

*(Participants selected either Strange or Not strange.)*

**Q2.**  *“Do you feel a sense of familiarity with this facial expression?”*

*(Participants selected either Familiar or Unfamiliar.)*

After all dialogue sessions had been completed, participants answered a post-experiment questionnaire concerning their overall impressions and comments about the interaction. The entire experiment took approximately 60 min per participant.

This study was approved by the Ethics Committee of RIKEN (Wako 2022-18). The data were pseudonymized and managed using participant IDs. The original recordings were retained to allow for potential additional analyses and are stored securely in locked storage with restricted access.

### 3.6. Participants

Fourteen participants took part in the experiment (seven male and seven female). Their ages ranged from 21 to 41 years, with a mean age of 30.6 years, and all participants were Japanese speakers. Two sessions were planned for each participant. However, due to system problems and experimental time constraints, 24 sessions were ultimately included in the analysis.

### 3.7. Data Processing and Statistical Analysis

#### 3.7.1. Analysis Data and Time Windows

The time at which each facial expression command was sent to the robot was defined as the reference time (t=0). The subsequent 2.5 s period was divided into five 0.5-s time windows: 0–0.5 s, 0.5–1.0 s, 1.0–1.5 s, 1.5–2.0 s, and 2.0–2.5 s. Referring to previous eye-tracking studies [47,48] and considering the time required for robot communication and expression generation, we set the total analysis period to 2.5 s. The 0.5-s window width was selected with future real-time HRI applications in mind, balancing sensitivity to short-term behavioral changes with robustness to temporal variability and measurement noise. Expression events were excluded if another facial-expression event occurred within the subsequent 2.5 s. A severe dialogue breakdown occurred in one session, and the affected segment was excluded from both the gaze and facial behavior analyses. No other sessions contained dialogue breakdowns that disrupted the continuity of the interaction.

For the gaze data, the two-dimensional gaze coordinates obtained from the eye tracker were mapped onto the participant’s first-person scene video. Nikola’s face was detected in each frame using MediaPipe Face Landmarker (Google, Mountain View, CA, USA) [49] (Figure 3). The detected facial landmarks were used to define the eye and mouth regions, with reference to previous eye-tracking research with a humanoid robot [50]. Each gaze sample was then assigned to one of five categories: the eye region, the mouth region, another area of the face, outside the face, or no face detected. Each gaze label was assumed to remain valid until the next gaze sample. Temporary gaps in gaze tracking could occur because of blinks, eye closure, or transient tracking loss. Expression events containing a gap of 0.5 s or longer were excluded, and only events with valid gaze data across all five time windows were retained. For each time window, gaze proportions for the robot’s eyes, mouth, and areas outside the face were calculated by dividing the corresponding gaze durations by the window duration. The gaze data were included regardless of the robot’s speaking state. After preprocessing, 677 expression events were included in the gaze analysis.

For facial behavior, four facial blendshape coefficients obtained from each video frame using MediaPipe Face Landmarker were analyzed: mouthSmileLeft, mouthSmileRight, mouthLowerDownLeft, and mouthLowerDownRight. These coefficients were selected because they capture upward and downward mouth movements associated with affective facial expressions and exhibited relatively large maximum values in the recorded data. In addition, because participants wore an eye tracker, the periocular region was occluded, making reliable analysis of eye- and eyebrow-related blendshapes difficult. Video was recorded at 30 fps, corresponding to 15 frames per 0.5-s time window. The value of each blendshape coefficient was averaged within each time window. The participant’s face was successfully detected in all frames of the expression events included in the analysis. To minimize the influence of participants’ own speech-related mouth movements, facial behavior was analyzed only while the robot was speaking. After preprocessing, 566 expression events were included in the facial behavior analysis.

#### 3.7.2. Statistical Analysis

For each participant, values were averaged across expression events separately for each time window and response category, and these participant-level means were used for statistical analysis. For Q1 and Q2, the two response categories were compared for each time window and behavioral measure using two-sided Wilcoxon signed-rank tests. Only participants with data for both response categories were included in the corresponding paired analyses. The gaze analyses included 13 participants for Q1 and 12 for Q2, whereas the facial behavior analyses included 12 participants for Q1 and 13 for Q2. The numbers differed because the different exclusion criteria described above were applied to the gaze and facial behavior data (Figure 4).

Temporal changes were additionally examined by calculating the difference between adjacent time windows for each participant and response category, defined as the value in the later window minus that in the preceding window. This resulted in four temporal changes: 0–0.5 to 0.5–1.0 s, 0.5–1.0 to 1.0–1.5 s, 1.0–1.5 to 1.5–2.0 s, and 1.5–2.0 to 2.0–2.5 s. For each transition and behavioral measure, the amount of change was compared between the two response categories using a two-sided Wilcoxon signed-rank test. Statistical significance was defined as p<0.05. Effect sizes for Wilcoxon signed-rank tests were calculated using the matched-pairs rank-biserial correlation ($r_{rb}$). Since this study was exploratory and no specific hypotheses were defined a priori, the analyses were intended to identify potential behavioral patterns rather than to provide confirmatory statistical evidence. Accordingly, uncorrected *p*-values are reported and should be interpreted as exploratory.

## 4. Results

Gaze proportions are reported as percentages, and their changes are reported in percentage points. Blendshape coefficients range from 0 to 1, with higher values indicating greater activation. Values are reported in the format of the median (mean ± standard error of the mean (SEM)). In figures, values are shown as the mean ± SEM for easier comparison.

### 4.1. Gaze Allocation

#### 4.1.1. Q1: Perceived Strangeness

The proportion of gaze directed to the mouth was higher for expressions rated as not strange than for those rated as strange at 0.5–1.0 s (W=8, p=0.006, $r_{rb}=0.824$). The value was 1.61 (3.00 ± 0.93) for expressions rated as strange and 7.51 (8.12 ± 1.90) for those rated as not strange. The same pattern was observed at 1.0–1.5 s (W=6, p=0.003, $r_{rb}=0.868$). Mouth-directed gaze was 0.85 (2.91 ± 1.24) for expressions rated as strange and 4.33 (7.68 ± 2.08) for those rated as not strange.

The change in mouth-directed gaze from 0–0.5 to 0.5–1.0 s also differed between ratings (W=7, p=0.005, $r_{rb}=0.846$). The change was −1.67 (−3.69±1.98) for expressions rated as strange and +3.44 (+3.56±0.92) for those rated as not strange.

No other comparisons among the three gaze measures were significant for Q1. The results of the gaze analysis for Q1 are shown in Figure 5a.

#### 4.1.2. Q2: Familiarity

No significant differences were observed in the gaze proportions within individual time windows. For changes between adjacent time windows, however, eye-directed gaze differed between ratings from 0.5–1.0 to 1.0–1.5 s (W=10, p=0.021, $r_{rb}=0.744$). The change was −3.56 (−3.78±1.81) for expressions rated as unfamiliar and +1.90 (+2.31±1.26) for those rated as familiar. Over the same interval, the change in gaze outside the face also differed between ratings (W=7, p=0.009, $r_{rb}=-0.821$). The change was +0.52 (+1.56±1.47) for expressions rated as unfamiliar and −2.06 (−2.74±1.42) for those rated as familiar.

No other comparisons among the three gaze measures were significant for Q2. The results of the gaze analysis for Q2 are shown in Figure 5b.

### 4.2. Facial Blendshapes

#### 4.2.1. Q1: Perceived Strangeness

At 1.0–1.5 s, mouthSmileLeft was higher for expressions rated as strange than for those rated as not strange (W=9, p=0.016, $r_{rb}=-0.769$). The value was 0.079 (0.132 ± 0.041) for expressions rated as strange and 0.040 (0.077 ± 0.032) for those rated as not strange. mouthSmileRight showed the same pattern (W=9, p=0.016, $r_{rb}=-0.769$). The value was 0.099 (0.177 ± 0.057) for expressions rated as strange and 0.054 (0.106 ± 0.043) for those rated as not strange.

At 2.0–2.5 s, all four blendshape coefficients were significantly higher for expressions rated as strange than for those rated as not strange. The values for strange and not-strange expressions were 0.080 (0.132 ± 0.042) and 0.043 (0.085 ± 0.033) for mouthSmileLeft (W=9, p=0.016, $r_{rb}=-0.769$), 0.092 (0.175 ± 0.058) and 0.054 (0.115 ± 0.047) for mouthSmileRight (W=8, p=0.012, $r_{rb}=-0.795$), 0.034 (0.062 ± 0.022) and 0.019 (0.035 ± 0.011) for mouthLowerDownLeft (W=10, p=0.021, $r_{rb}=-0.744$), and 0.034 (0.067 ± 0.023) and 0.021 (0.036 ± 0.012) for mouthLowerDownRight (W=11, p=0.027, $r_{rb}=-0.718$), respectively.

No other significant differences were observed within individual time windows or in changes between adjacent time windows for Q1. The results of the facial behavior analysis for Q1 are shown in Figure 6a.

#### 4.2.2. Q2: Familiarity

None of the four blendshape coefficients showed significant differences within individual time windows or between adjacent windows. The results of the facial behavior analysis for Q2 are shown in Figure 6b.

## 5. Discussion

### 5.1. Mouth-Directed Gaze and Perceived Strangeness

From 0–0.5 s to 0.5–1.0 s after the facial-expression command, mouth-directed gaze increased for expressions rated as not strange, whereas it decreased for those rated as strange. In addition, the proportion of mouth-directed gaze was lower for expressions rated as strange than for those rated as not strange at both 0.5–1.0 s and 1.0–1.5 s. This pattern suggests that gaze behavior toward the mouth began to differ relatively early depending on whether the expression was perceived as strange.

One possible explanation is that participants tended to avert their gaze from a visually salient part of the expression when they experienced perceptual discomfort or a mismatch between the expression and the conversational context. A relationship between perceived uncanniness and gaze aversion has also been reported in HRI [25].

The mouth may have been particularly informative in this process. Previous studies of human facial expressions have shown that the mouth is a particularly informative region for discriminating facial expressions [51] and that subtle differences in mouth configuration can affect facial-expression perception [52]. For Nikola in particular, the mouth is a visually salient region because its shape changes substantially through the movement of multiple facial actuators. Inconsistencies in shape or motion may therefore be particularly noticeable around the mouth. When such inconsistencies were perceived, participants may have shifted their gaze away from this region rather than continuing to attend to it. Taken together, the early decrease in mouth-directed gaze for expressions perceived as strange may reflect gaze aversion associated with perceptual discomfort or a mismatch between the facial expression and the conversational context.

### 5.2. Facial Responses to Perceived Strangeness

In addition to the differences in gaze behavior, expressions perceived as strange were associated with greater mouth-related facial activity in participants. At 1.0–1.5 s, mouthSmileLeft and mouthSmileRight were higher for expressions rated as strange, and at 2.0–2.5 s, all four mouth-related blendshapes showed higher values for strange than for not-strange expressions. These results suggest that perceived strangeness was reflected not only in where participants looked, but also in their own facial behavior.

One possible interpretation of these responses involves sensorimotor simulation during facial-expression perception. When observing another person’s facial expression, observers can partially activate sensorimotor representations corresponding to the observed facial movements, which may contribute to facial-expression recognition and interpretation [53]. Such responses can also vary depending on how the expression is subjectively perceived. Nakamura and Tanaka found that facial mimicry differed depending on whether participants perceived the same ambiguous facial stimulus as happiness or anger, suggesting that subjective interpretation can modulate facial responses [54]. The observer’s own facial activity may also contribute to expression recognition. Wingenbach et al. found that facial muscle activity incongruent with an observed expression reduced recognition accuracy, particularly when the manipulated region overlapped with a region important for recognizing the expression [55]. These findings suggest that facial movements are involved in the perception and interpretation of observed expressions. From this perspective, the greater mouth-related facial activity observed for expressions perceived as strange may reflect sensorimotor processing associated with participants’ perception of those expressions.

### 5.3. Gaze Dynamics and Perceived Familiarity

The Q2 results concerned temporal redistribution rather than overall gaze level. From 0.5–1.0 to 1.0–1.5 s, gaze behavior changed in opposite directions between the two ratings. For familiar expressions, eye-directed gaze increased while gaze outside the face decreased. In contrast, for unfamiliar expressions, eye-directed gaze decreased while gaze outside the face increased.

This temporal pattern may reflect a shift from initial expression processing to sustained attention toward the interaction partner. Previous studies have shown that basic emotional expressions can be identified within several hundred milliseconds of stimulus presentation [56]. Given the time required for Nikola’s facial expression to be fully formed, the gaze changes observed around 1.0 s may have occurred after the initial evaluation of the expression. At this stage, maintained eye-directed gaze may reflect continued social attention toward the interaction partner. This interpretation is consistent with previous HRI research showing that greater mutual gaze is associated with higher perceived likability and warmth [25]. Accordingly, maintained eye-directed gaze after the initial evaluation may reflect sustained social engagement and more positive impressions of the interaction partner.

### 5.4. Limitations

Several limitations should be noted. First, free conversation reduced experimental control. This setting was chosen to reflect the intended use of the system in real-world HRI. However, the conversations tended to be casual and positive in tone, and the facial expressions generated by the robot were consequently concentrated in the neutral-to-positive range. This imbalance in expression types may have influenced the observed gaze patterns, as previous studies have shown that gaze allocation across facial regions varies depending on the emotional expression being observed [57,58,59]. Participants’ facial behavior may likewise vary depending on the observed expression, with different emotional expressions eliciting distinct patterns of facial movement [23,35,36]. Future studies should consider interaction strategies that naturally produce a broader range of emotional contexts, including those in which negative expressions, such as those of sadness or anger, can occur. In addition, controlled experimental designs should also be considered. For example, experimentally introducing incongruent combinations of speech and facial expressions would make it possible to examine subjective ratings of strangeness in relation to an objectively defined mismatch between verbal and facial cues. Nevertheless, neutral-to-positive expressions are likely to occur frequently in practical dialogue-based service interactions, and the present findings may therefore still provide useful insights for such applications.

Second, eye- and eyebrow-related facial actions were not analyzed in this study because the wearable eye tracker occluded the periocular region. These facial actions may also provide important information about participants’ responses to robot facial expressions. Future studies should therefore examine a broader range of facial movements by conducting experiments focusing on facial responses without a wearable eye tracker, thereby allowing unobstructed measurement of the entire face.

Third, the experiment was conducted with only one android robot. Previous research on human faces has shown that gaze allocation can vary depending on the gender of the face being viewed. Among opposite-gender observers, greater attention to the mouth of female faces and to the eyes and hair of male faces has been associated with higher attractiveness ratings [60]. This suggests that the appearance and characteristics of an interaction partner may influence how visual attention is distributed across facial regions. Future studies should therefore investigate these relationships across a wider variety of robots with different appearances and characteristics.

Finally, the sample size was relatively small, and the participants were drawn from a limited cultural background. Considering individual differences in gaze behavior during face perception [61], the present sample may not capture the full range of behavioral and perceptual variability. Cultural background may also influence how facial expressions are interpreted and how visual attention is distributed during social interaction. A larger and more diverse sample will be necessary to assess the generalizability of these relationships.

Despite these limitations, this study provides initial evidence that subjective perceptions of android facial expressions are reflected in gaze and facial responses during natural interaction. These findings may contribute to the development of adaptive HRI systems that respond to how users perceive robot facial expressions.

## 6. Conclusions

This study investigated how subjective perceptions of an android robot’s facial expressions during face-to-face conversation were associated with participants’ gaze and facial behaviors. Expressions perceived as strange were associated with reduced mouth-directed gaze shortly after facial-expression command transmission and with greater mouth-related facial activity at later time points. Perceived familiarity was associated with differences in subsequent gaze dynamics, particularly in the redistribution of attention between the eyes and areas outside the face. These findings suggest that subjective differences in the perception of individual robot facial expressions can be reflected in short-term nonverbal behavior during ongoing interaction. The results provide exploratory observations that can guide future studies of behavioral cues for adaptive facial-expression generation in real-time HRI. In future work, we aim to develop interaction strategies that incorporate these cues to better respond to individual users.

## Figures and Tables

**Figure 1:**
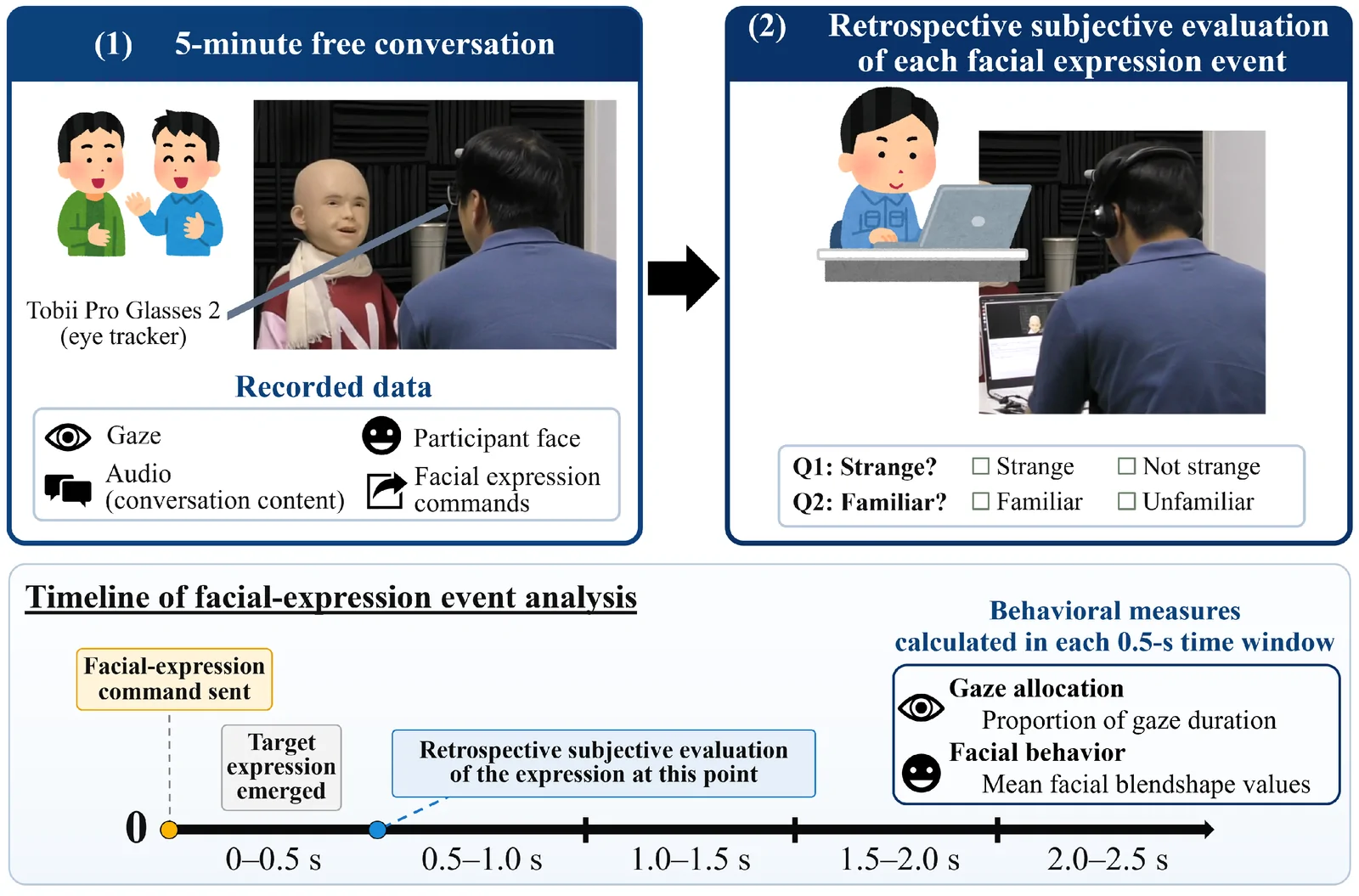
Overview of the experimental procedure and analysis.

**Figure 2:**
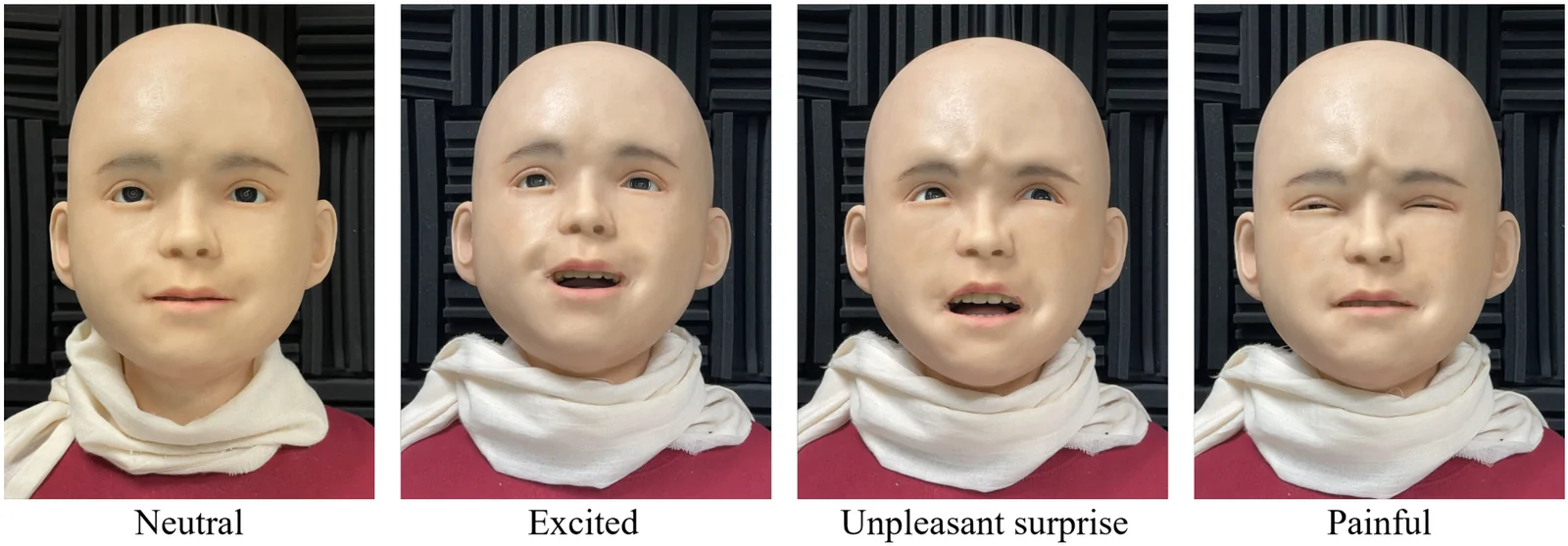
Examples of the variety of facial expressions performed by Nikola.

**Figure 3:**
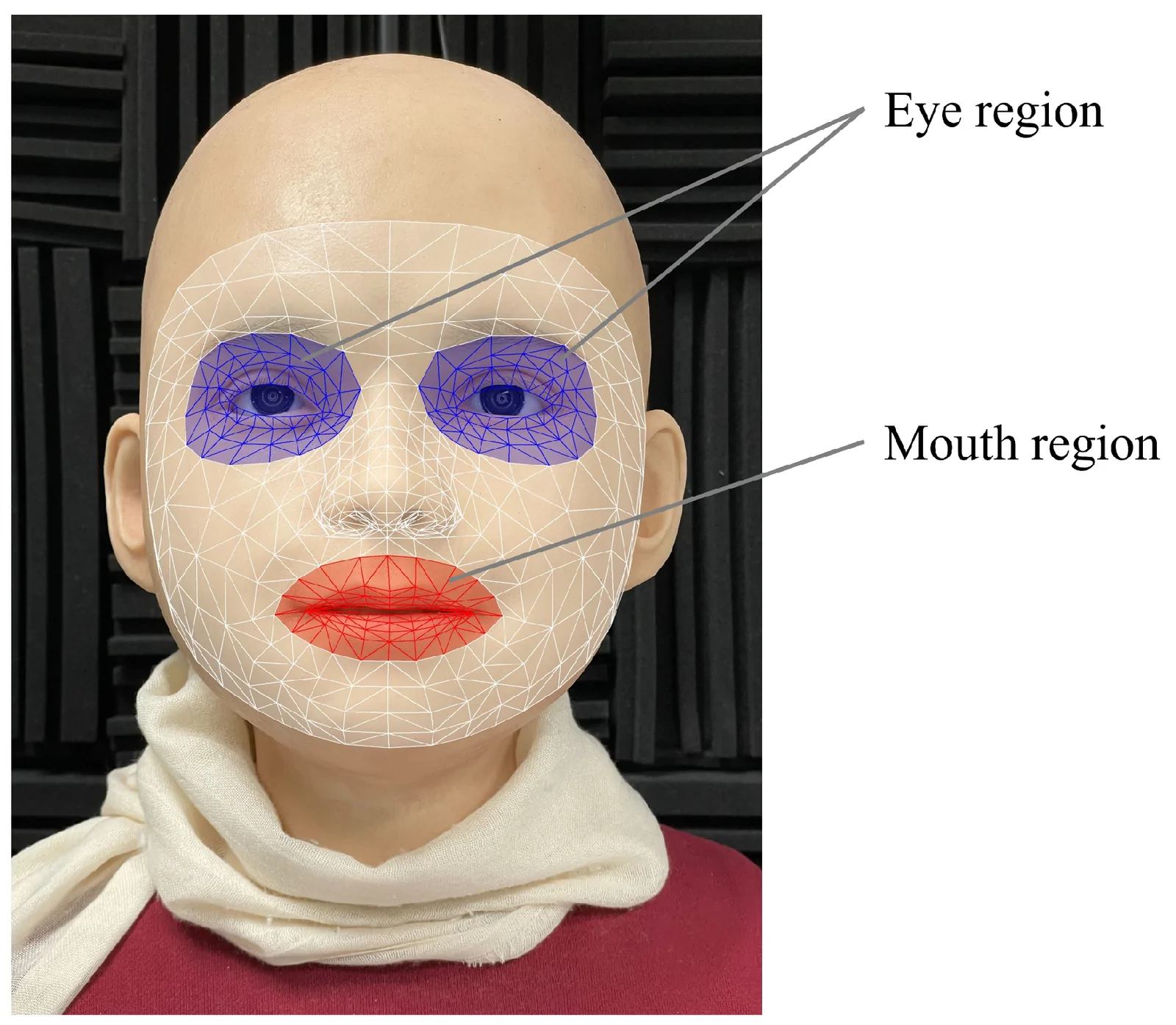
Definition of the eye and mouth regions used for gaze classification.

**Figure 4:**
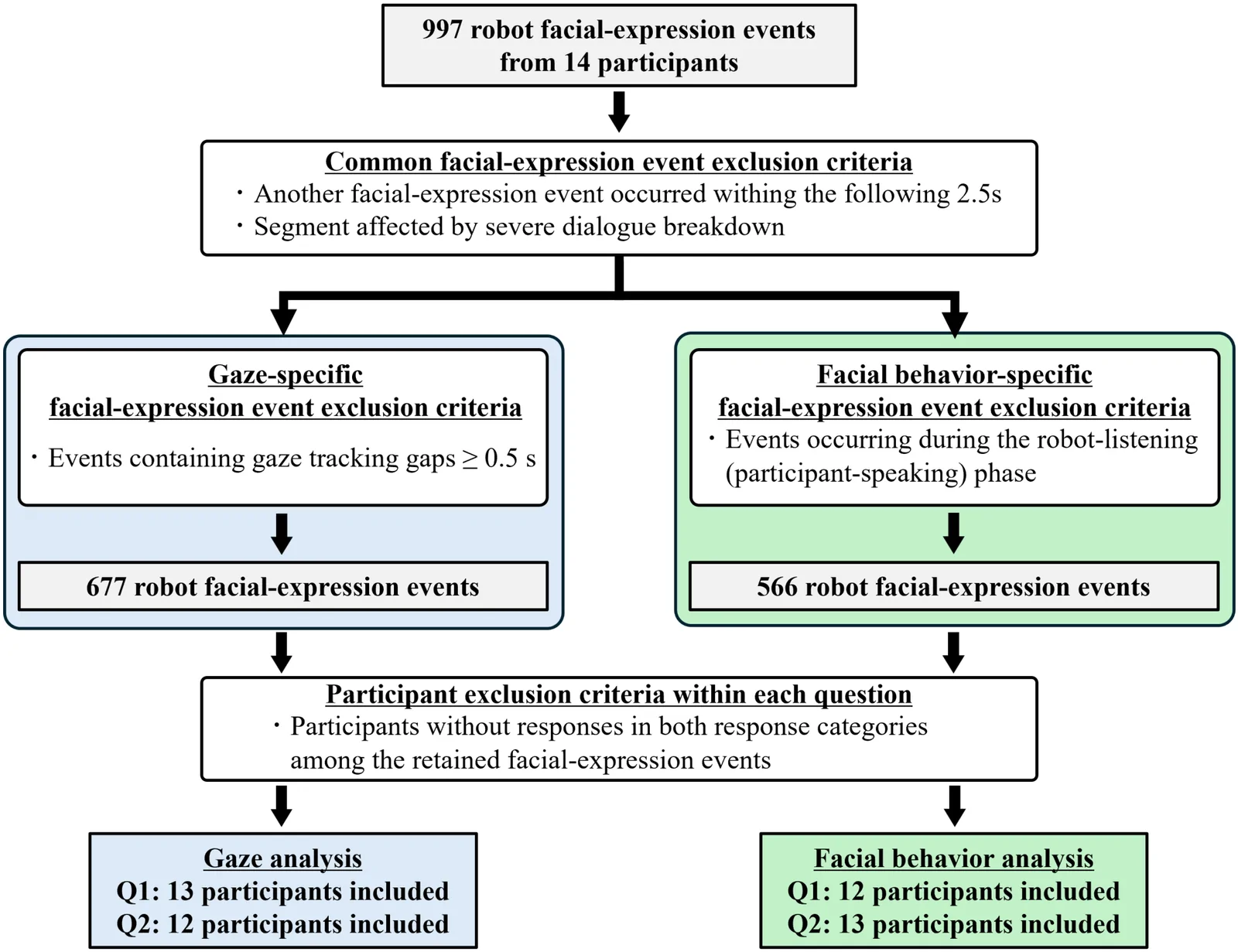
Flowchart of facial-expression event and participant exclusion procedures for the gaze and facial behavior analyses. Blue and green backgrounds indicate the gaze and facial behavior analyses, respectively.

**Figure 5:**
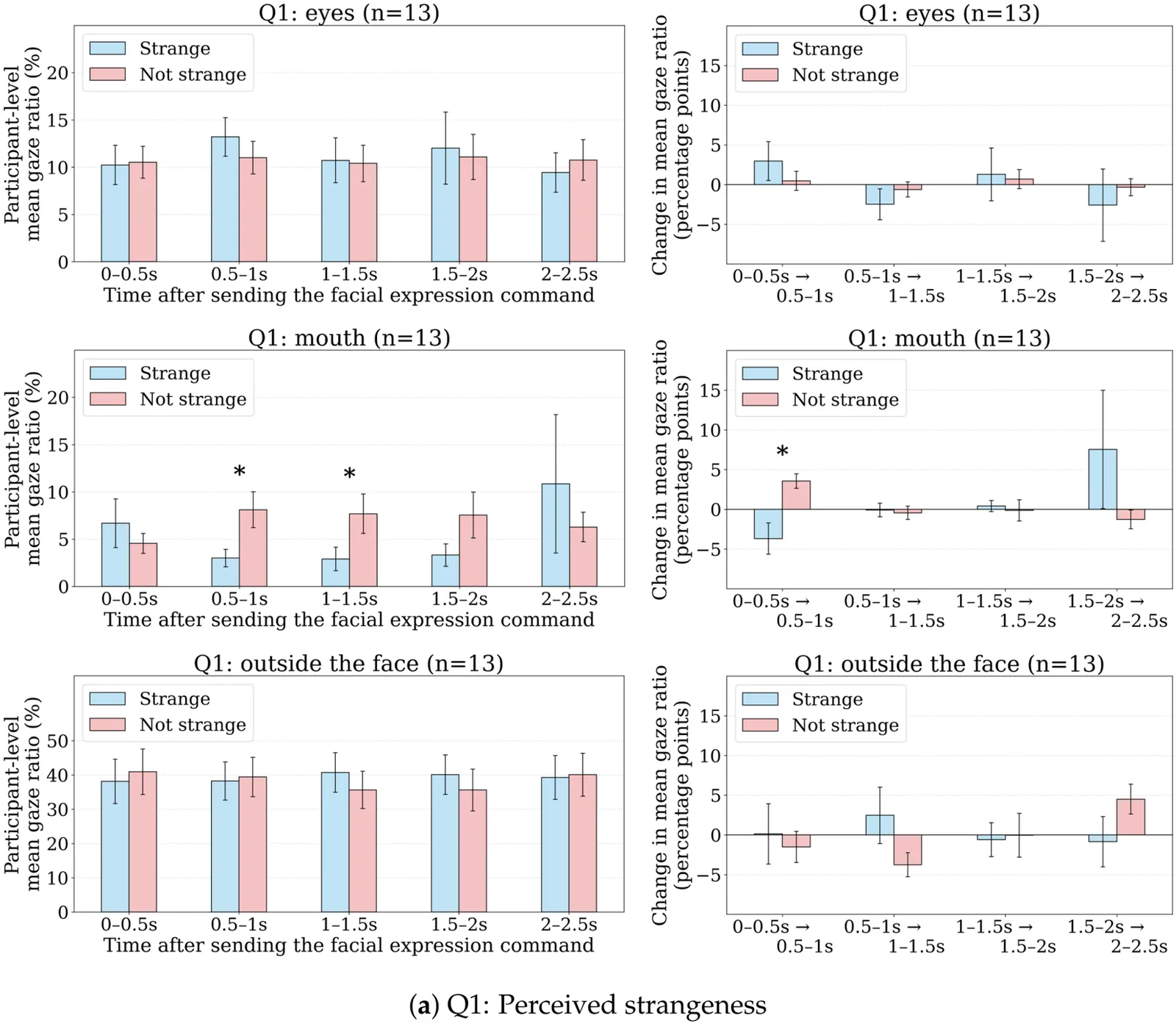

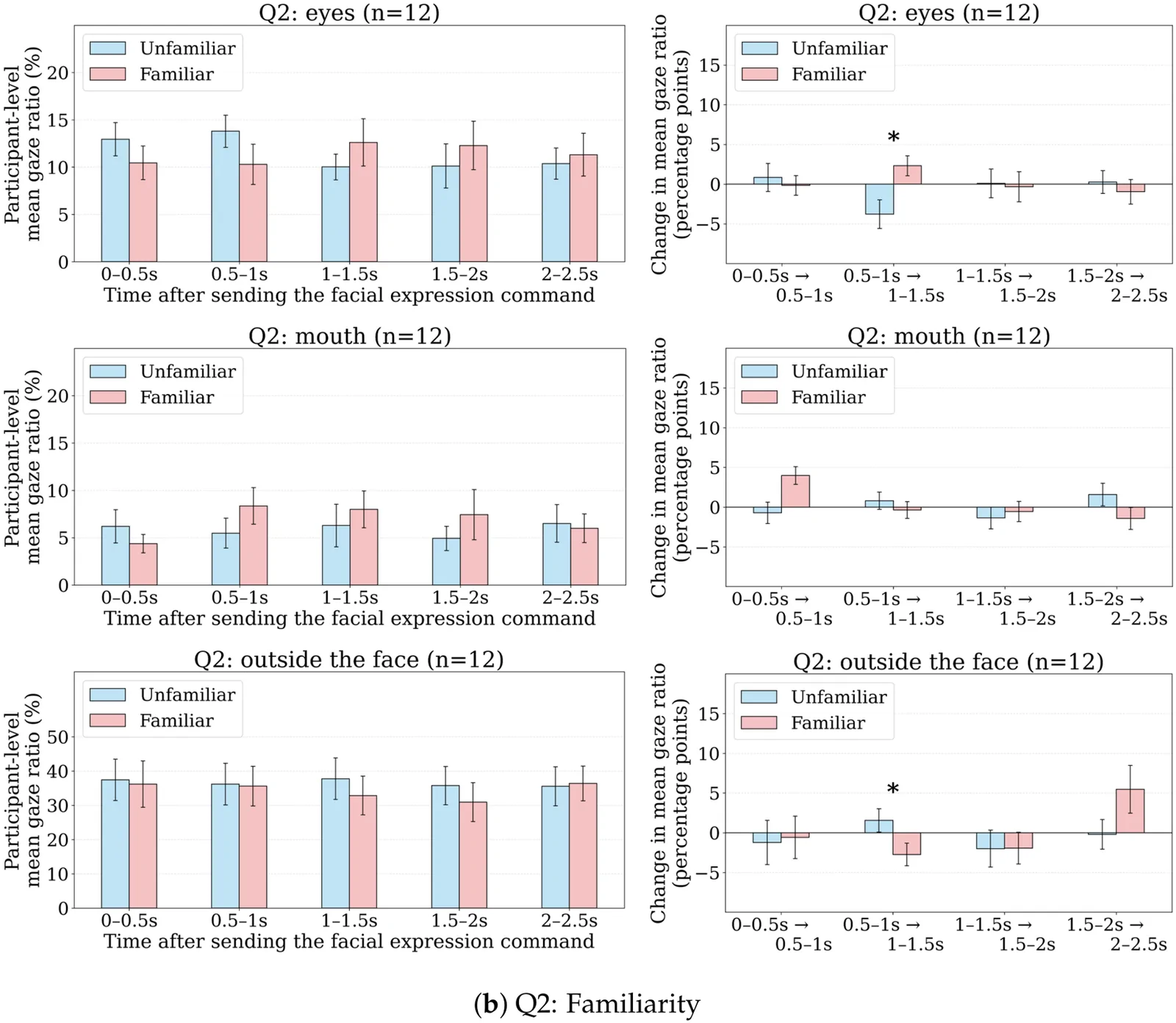
Gaze behavior according to subjective impressions. The left column shows gaze proportions in each time window, and the right column shows changes across adjacent time windows. *: p<0.05.

**Figure 6:**
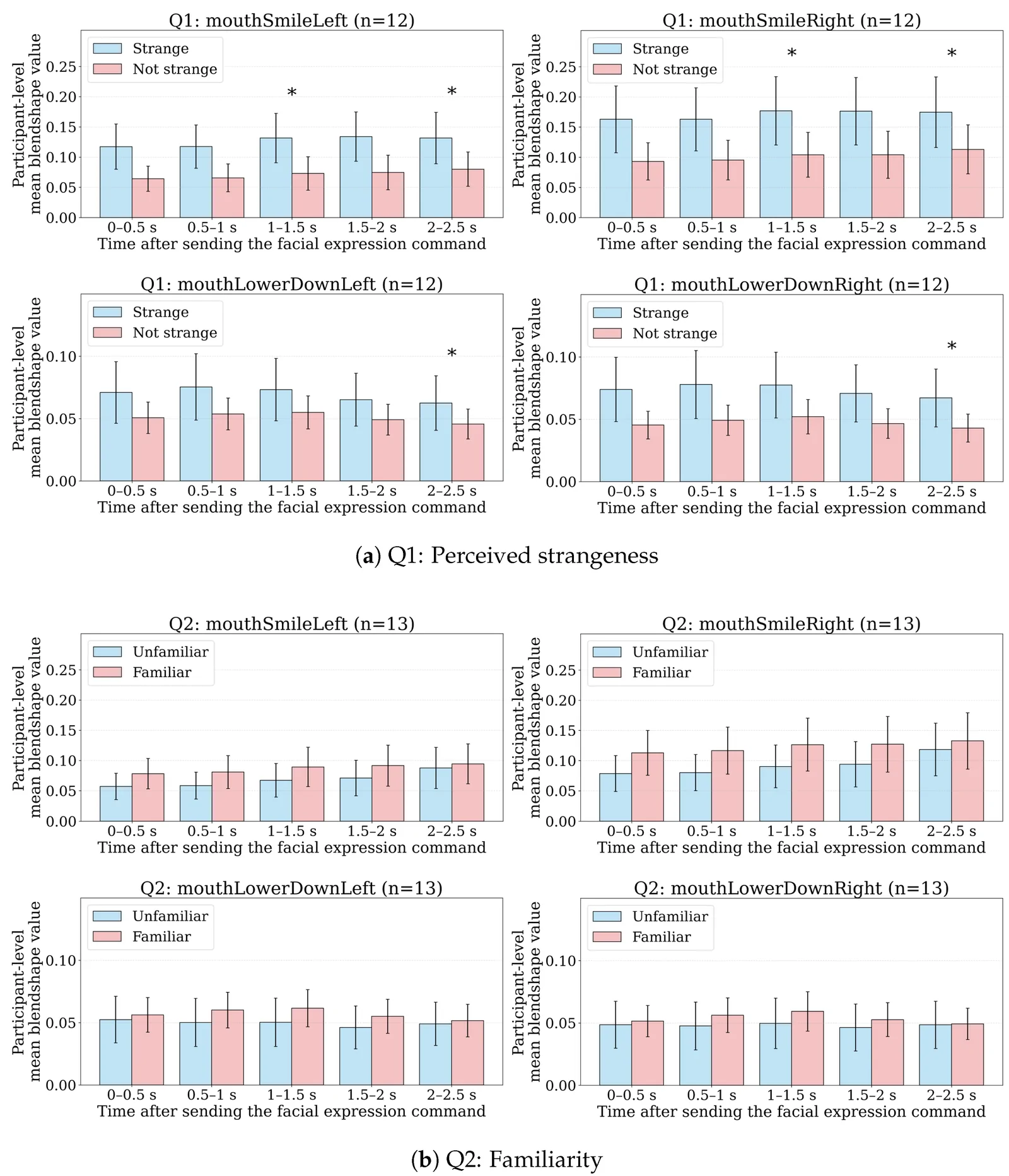
Facial blendshapes in each time window according to subjective impressions. *: p<0.05.

## Data Availability

The original contributions presented in the study are included in the article. Further inquiries can be directed to the corresponding author.
